# Brain Age Prediction in Generalized Anxiety Disorder using a Convolutional Neural Network

**DOI:** 10.21203/rs.3.rs-6866544/v1

**Published:** 2025-07-01

**Authors:** Corey Richier, André Zugman, Anita Harrewijn, Elise M. Cardinale, Parmis Khosravi, Moji Aghajani, Willem B. Bruin, Kevin Hilbert, Narcis Cardoner, Daniel Porta-Casteràs, Marta Cano, Savannah Gosnell, Ramiro Salas, Andrea P. Jackowski, Pedro M. Pan, Giovanni A. Salum, Karina S. Blair, James R. Blair, Mohammed R. Milad, Katie L. Burkhouse, K. Luan Phan, Heidi K. Schroeder, Jeffrey R. Strawn, Katja Beesdo-Baum, Neda Jahanshad, Sophia I. Thomopoulos, Jared A. Nielsen, Jordan W. Smoller, Jair C. Soares, Benson Mwangi, Mon-Ju Wu, Giovana B. Zunta-Soares, Michal Assaf, Gretchen J. Diefenbach, Paolo Brambilla, Eleonora Maggioni, David Hofmann, Thomas Straube, Carmen Andreescu, Rebecca B. Price, Gisele G. Manfro, Federica Agosta, Elisa Canu, Camilla Cividini, Massimo Filippi, Milutin Kostić, Ana Munjiza Jovanovic, Brenda Benson, Gabrielle F. Freitag, Ellen Leibenluft, Grace V. Ringlein, Kathryn Werwath, Hannah Zwiebel, Hans J. Grabe, Sandra Van der Auwera, Katharina Wittfeld, Henry Völzke, Robin Bülow, Nicholas L. Balderston, Monique Ernst, Lilianne R. Mujica-Parodi, Helena van Nieuwenhuizen, Hugo D. Critchley, Elena Makovac, Matteo Mancini, Frances Meeten, Cristina Ottaviani, Gregory A. Fonzo, Martin P. Paulus, Murray B. Stein, Raquel E. Gur, Ruben C. Gur, Antonia N. Kaczkurkin, Bart Larsen, Theodore D. Satterthwaite, Jennifer Harper, Michael T. Perino, Chad M. Sylvester, Qiongru Yu, Patrick McClure, Francisco Pereira, Ulrike Lueken, Dick J. Veltman, Paul M. Thompson, Nynke A. Groenewold, Janna Marie Bas-Hoogendam, Dan J. Stein, Nic J. A. Van der Wee, Anderson M. Winkler, Daniel S. Pine, Chelsea K. Sawyers

**Affiliations:** 1. Department of Psychology, University of Illinois at Urbana-Champaign, Urbana, IL, USA; 2. Emotion and Development Branch, National Institute of Mental Health, Bethesda, MD, USA; 3. Department of Psychology, Education and Child Studies, Erasmus University Rotterdam, Rotterdam, The Netherlands; 4. Department of Psychology, The Catholic University of America; 5. Leiden University, Institute of Education & Child Studies, Section Forensic Family & Youth Care; 6. Department of Psychology, HMU Health and Medical University Erfurt, Erfurt, Germany; 7. Sant Pau Mental Health Research Group, Institut de Recerca Sant Pau (IR SANT PAU), Barcelona, Spain; 8. Centro de Investigación Biomédica En Red en Salud Mental (CIBERSAM), Instituto de Salud Carlos III, Madrid, Spain; 9. Instituto de Salud Carlos III (ISCIII), Madrid, Spain; 10. Menninger Department of Psychiatry and Behavioral Sciences, Baylor College of Medicine, Houston, TX, USA; 11. LiNC, Department of Psychiatry, Federal University of São Paulo, São Paulo, Brazil; 12. Section on Negative Affect and Social Processes, Hospital de Clínicas de Porto Alegre, Universidade Federal do Rio Grande do Sul, Porto Alegre, Brazil; 13. Center for Neurobehavioral Research, Boys Town National Research Hospital, Boys Town, NE, USA; 14. Child and Adolescent Mental Health Center, Copenhagen University Hospital – Mental Health Services CPH, Copenhagen, Denmark; 15. Department of Clinical Medicine, Faculty of Health and Medical Sciences, University of Copenhagen, Denmark; 16. Department of Psychiatry, NYU School of Medicine, New York University, New York, NY, USA; 17. Department of Psychology, Penn State University, University Park, PA, USA; 18. Department of Psychiatry and Behavioral Health, The Ohio State University, Columbus, OH, USA; 19. Department of Psychiatry & Behavioral Neuroscience, University of Cincinnati, Cincinnati, OH, USA; 20. Behavioral Epidemiology, Institute of Clinical Psychology and Psychotherapy, Technische Universität Dresden, Dresden, Germany; 21. Imaging Genetics Center, Mark and Mary Stevens Neuroimaging and Informatics Institute, Keck School of Medicine, University of Southern California, Marina del Rey, CA, USA; 22. Center for Brain Science & Department of Psychology, Harvard University, Cambridge, MA, USA; 23. Department of Psychiatry, Massachusetts General Hospital, Boston, MA, USA; 24. Psychology Department & Neuroscience Center, Brigham Young University; 25. Center Of Excellence On Mood Disorders, Louis A. Faillace, MD, Department of Psychiatry and Behavioral Sciences, The University of Texas Health Science Center at Houston, Houston, TX, USA; 26. Olin Neuropsychiatry Research Center, Institute of Living, Hartford Hospital, Hartford, CT, USA; 27. Department of Psychiatry, Yale School of Medicine, New Haven, CT, USA; 28. Anxiety Disorders Center, Institute of Living, Hartford Hospital, Hartford, CT, USA; 29. Department of Neurosciences and Mental Health, Fondazione IRCCS Ca’ Granda Ospedale Maggiore Policlinico, Milan, Italy; 30. Department of Pathophysiology and Transplantation, University of Milan, Milan, Italy; 31. Institute of Medical Psychology and Systems Neuroscience, University of Muenster, Muenster, Germany; 32. Department of Psychiatry, University of Pittsburgh, Pittsburgh, PA, USA; 33. Department Psychology, University of Pittsburgh, Pittsburgh, PA, USA; 34. Anxiety Disorder Program, Hospital de Clínicas de Porto Alegre, Department of Psychiatry, Federal University of Rio Grande do Sul, Porto Alegre, Brazil; 35. Neuroimaging Research Unit, Institute of Experimental Neurology, Division of Neuroscience, IRCCS San Raffaele Scientific Institute, Milan, Italy; 36. Vita-Salute San Raffaele University, Milan, Italy; 37. Neurology Unit, IRCCS San Raffaele Scientific Institute, Milan, Italy; 38. Neurophysiology Unit, IRCCS San Raffaele Scientific Institute, Milan, Italy; 39. Neurorehabilitation Unit, IRCCS San Raffaele Scientific Institute, Milan, Italy; 40. Institute of Mental Health, University of Belgrade, Belgrade, Serbia; 41. Department of Psychiatry, School of Medicine, University of Belgrade, Belgrade, Serbia; 42. Department of Biostatistics, Johns Hopkins Bloomberg School of Public Health, Baltimore, MD, USA; 43. Psychiatry and Behavioral Sciences, Stanford University School of Medicine, Stanford, CA, USA; 44. Department of Psychiatry and Psychotherapy, University Medicine Greifswald, Greifswald, Germany; 45. German Center for Neurodegenerative Diseases (DZNE), Site Rostock/Greifswald, Greifswald, Germany; 46. Institute for Community Medicine, University Medicine Greifswald, Greifswald, Germany; 47. Institute for Diagnostic Radiology and Neuroradiology, University Medicine Greifswald, Greifswald, Germany; 48. Center for Neuromodulation in Depression and Stress, University of Pennsylvania, Philadelphia, PA, USA; 49. Section on Neurobiology of Fear and Anxiety, National Institute of Mental Health, Bethesda, MD, USA; 50. Department of Biomedical Engineering, Stony Brook University, Stony Brook, NY, USA; 51. Department of Physics, Stony Brook University, Stony Brook, NY, USA; 52. Department of Neuroscience, Brighton and Sussex Medical School, University of Sussex, Brighton, UK; 53. Department of Psychology, Brunel University London; 54. “Enrico Fermi” Research Center, Rome, Italy; 55. Department of Psychology, Institute of Psychiatry, Psychology and Neuroscience, King’s College London, London, UK; 56. Department of Psychology, Sapienza University of Rome, Rome, Italy; 57. IRCCS Santa Lucia Foundation; 58. Department of Psychiatry and Behavioral Sciences, The University of Texas at Austin Dell Medical School, Austin, TX, USA; 59. Laureate Institute for Brain Research, Tulsa, OK, USA; 60. Department of Psychiatry, School of Medicine and Herbert Wertheim School of Public Health, University of California, San Diego, La Jolla, CA, USA; 61. Department of Psychiatry, University of Pennsylvania, Philadelphia, PA, USA; 62. Department of Psychology, Vanderbilt University, Nashville, TN, USA; 63. Department of Pediatrics, University of Minnesota, Minneapolis, MN, USA; 64. Department of Psychiatry, Washington University, St. Louis, MO, USA; 65. Machine Learning Core, National Institute of Mental Health, Bethesda, MD, USA; 66. Department of Psychology, Humboldt-Universität zu Berlin, Berlin, Germany; German Center for Mental Health (DZPG), partner site Berlin-Potsdam; 67. Department of Psychiatry, Amsterdam UMC, location VUMC, Amsterdam, The Netherlands; 68. Department of Psychiatry & Neuroscience Institute, University of Cape Town, Cape Town, South Africa; 69. Department of Psychiatry, Leiden University Medical Center, Leiden, The Netherlands; 70. Department of Developmental and Educational Psychology, Institute of Psychology, Leiden University, Leiden, The Netherlands; 71. Leiden Institute for Brain and Cognition, Leiden, The Netherlands; 72. South African Medical Research Council Unit on Risk & Resilience in Mental Disorders, Department of Psychiatry & Neuroscience Institute, University of Cape Town, Cape Town, South Africa; 73. University of Texas Rio Grande Valley, Brownsville, TX, USA; 74. Division of Child and Adolescent Psychiatry, Department of Psychiatry, Virginia Commonwealth University

## Abstract

Higher predicted brain age difference has been associated with several psychiatric disorders. Generalized anxiety disorder (GAD) is associated with markers of accelerated aging. In this study, we determined brain predicted age difference (PAD) in individuals with GAD and healthy controls (HC) as well as group differences in PAD variability using voxel-wise structural MRI. The training dataset included 3,511 controls, and the testing dataset included 1,595 individuals with GAD and 4,552 HC from the ENIGMA-Anxiety GAD Working Group. A convolutional neural network model using four input modalities per subject and a model ensemble approach was used to predict brain age. The PAD was then calculated by subtracting chronological age. Model performance was consistent with other image-based brain age prediction models with similar accuracy across the training set (mean absolute error (MAE) = 2.95 years) and HC in the testing set (MAE = 2.94). We found no evidence of accelerated brain aging in individuals with GAD, though we did find evidence for greater variation in PAD for individuals with GAD (Levene’s test: W = 442.98, *p* < .001) and evidence for greater variability in PAD of those with GAD over 25 years of age. No relationships between PAD and clinical or demographic measures were found. To conclude, using large training and testing samples, the study found no significant association between GAD and PAD, although individuals with GAD had greater heterogeneity in brain-predicted age.

## Introduction

Generalized anxiety disorder (GAD) is one of the most prevalent forms of psychiatric disorders, having a lifetime prevalence of 6.2% [1–3]. GAD has a peak onset in late adolescence and early adulthood and high comorbidity rate [2–4]. In this study, we leverage available large imaging datasets to investigate whether GAD is associated with an individual’s predicted “brain age”. Brain age modeling uses neuroimaging data to estimate an individual’s chronological age, with deviations between predicted and actual age—referred to as the predicted age difference (PAD)—increasingly regarded as potential biomarkers of atypical neurodevelopment or neurodegeneration[5, 6]. These brain aging estimates may proceed faster or slower than would be expected. A positive predicted age difference (PAD) may indicate accelerated maturation, or an “older” brain than expected given a chronological age; conversely, a negative PAD may indicate a “younger” brain. Such investigations would be particularly relevant for GAD as individuals with this condition appear to have structural, functional, and cognitive differences [7–10], many of which occurring at critical phases of brain development, such as late childhood and adolescence

Higher brain PAD has been associated with a wide range of physical and psychological conditions including cognitive impairment [11, 12], traumatic brain injury [13], schizophrenia [14, 15], childhood adversity [16], and major depressive disorder [17]. More broadly, psychiatric disorders are associated with advanced brain age [17–20]. However, findings related to brain age and anxiety disorders are inconsistent. For example, one study found no relationship between dimensional measures of anxiety in youth, but no significant PAD [21]; however another study found a positive PAD of 2.91 years in adults (N=220) with anxiety disorders, including GAD, panic disorder (PD), and social anxiety disorder (SAD; [18]. Interestingly, this finding was only significant after accounting for medication use. More recently, in a study of individuals with SAD and GAD, adolescents with more severe GAD showed a higher PAD compared to controls, and adolescents with more severe SAD symptoms showed a lower PAD [22]. To date, no work has examined brain age in individuals with GAD as a distinct group.

Given unclear evidence regarding the association of brain age and anxiety disorders, we aim to evaluate PAD and assess its relation to the presence of GAD. To that end, we use a convolutional neural network (CNN) model to predict the age of each individual from their structural magnetic resonance images (MRI) and compute their PADs. CNNs are deep learning models for computer vision inspired by the brain’s visual cortex [23], and are commonly used to examine brain age [24–27] as they are well suited for multidimensional data. These models capture spatial relations and features in their input data and are sensitive to subject-level spatial information. This can be a strength relative to univariate models (voxelwise, vertex-wise, or feature-based), as CNNs can extract additional structural information from the image, encoded in spatial patterns. The nonlinear nature of deep learning models (including CNN) allows for detection of complex relations between the input data and the subject’s age. Moreover, in healthy individuals, CNN models produce more accurate age estimations than other modeling frameworks, such as linear models or other non-neural network machine learning methods [28].

Our study has two main hypotheses: first, that PAD would be greater in participants with GAD compared to healthy controls (HC); and that larger variance in PAD in GAD. Second, participants with GAD might have a greater prediction error by the CNN, thus leading to increased variance in the PAD for this group. Other brain age research has found greater variability in age predictions for clinical groups relative to individuals without psychopathology [17, 18, 29]. To assess the second hypothesis, we conduct a test to verify the equality of variance between subjects with and without GAD. This could reflect greater heterogeneity in individuals with GAD potentially derived from factors such as treatment and disease severity while controls would form a more homogeneous group. Likewise, this variance might change as a factor of age [30, 31]. Therefore, we also hypothesize that variance in age prediction will depend on age. As secondary goals we investigate if PAD will be related to medication, comorbidity, and age-by-diagnosis interaction. To address these questions, we use structural brain MRI data from the Enhancing Neuroimaging Genetics through Meta-Analysis (ENIGMA) Anxiety Working Group [32, 33]. This was the largest study to date investigating structural alterations between GAD and controls, and found no association between GAD and brain morphometry in a well-powered mega-analysis of 28 sites [33]. The present study builds on that work to examine if “brain age” might reveal structural differences in GAD that were not detected in the mass univariate analysis of the previous work. As PAD is estimated from a multivariate (CNN) model, it may have more sensitivity than mass-univariate testing as done in the previous ENIGMA-Anxiety GAD work. Moreover, previous work on other cohorts have shown different results when using “brain age” estimated from features obtained from FreeSurfer [21]. Previous work on brain PAD both within other ENIGMA working groups [17] and outside of ENIGMA [21, 22] have used a variety of machine learning methods to build and train brain-age prediction models. Based on the model accuracy findings from Jonnson et al. [34], showing the superiority of a model combining different measures obtained by T1-weighted scans, we build a new voxelwise CNN model rather than via feature extraction (e.g., from FreeSurfer) to limit the loss of information.

## Methods

### Sample Selection

The present analysis is a pre-registered (https://osf.io/4wtdy) mega-analysis [35] of structural brain MRI data, from an international multi-site sample. Our training sample of 3,511 individuals includes several publicly available neuroimaging samples of both pediatric and adult healthy controls, including the Human Connectome Project [36], Open Access Series of Imaging Studies 3 (OASIS-3; [37], Philadelphia Neurodevelopmental Cohort (PNC; [38], and the Pediatric Imaging, Neurocognition, and Genetics (PING) study [39]. The present analysis only used images from a single time point in studies with longitudinal data. OASIS-3 [37] images were randomly selected across participants across all three timepoints to provide a wider distribution of ages within the sample.

The clinical test sample consists of data from the ENIGMA Anxiety Working Group [35] with 6,147 subjects from 32 studies. This includes ABCD [40], CMI [41] and BHRC [42] as well as data provided from ENIGMA Anxiety participating sites. As above we only used a single time point for each study. In the case of ABCD and BHRC we only include the baseline data. The selection process for determining the final number of images used in the model is summarized in Supplemental Table 1, which illustrates the procedure for inclusion of participants. We excluded subjects due to failed processing, missing covariates (age, sex, diagnosis), comorbid psychosis, bipolar disorder or ASD, and subjects with no GAD diagnosis but any other psychiatric diagnosis.

### Neuroimaging data preprocessing

All data were run through the FSL voxel-based morphometry (VBM) pipeline [43, 44], providing voxelwise output [45]. Such output is amenable for use with CNNs, while still capturing between-subject variability on brain morphology. Structural T1-weighted MRI brain images were first standardized and underwent intensity normalization. Afterwards, non-brain tissue was removed. Following this, the T1-weighted images were bias corrected. Images were then segmented into gray and white matter maps, modulated, and smoothed. It has been demonstrated that an increased number of imaging modalities results in better prediction [34]; thus, for this analysis we used four images derived from the VBM pipeline: grey matter, white matter, T1-weighted image, and the image of the Jacobian of the non-linear transformation used to map the individual T1-weighted image to the reference standard space [46]; the latter provides a measure of stretches and shrinkages needed to non-linearly align each subject’s image to a standard space, in this case, the MNI152 [47].

No data harmonization step was implemented (e.g., ComBat; [48]) due to recent demonstrations that not applying site/scanner harmonization produces lower mean absolute error (MAE) for brain age prediction than applying harmonization methods [49]. After the VBM process was completed, images were converted to NumPy arrays [50] to increase speed of image loading, and were cropped from their original dimension of 91 × 109 × 91 to 75 × 88 × 78 to reduce the amount of empty space in the image surrounding the brain tissue.

### Quality control

Quality control procedures consisted of visual inspection to exclude any images that were misshapen or contained any other processing errors. Additionally, subjects who had errors in their covariate entries (see Supplemental Table 1) were excluded. After quality control, the remaining usable sample consisted of 8,063 HC and 1,595 participants with a diagnosis of GAD, generating a total sample size of 9,658. Tables 1 and 2 detail the number of participants included from each sample for the training and testing samples. Data use guidelines for all research sites were followed. Appropriate consent and assent procedures were obtained for all participants at all sites. Individuals with GAD had either a lifetime or present diagnosis of GAD. These diagnoses were established at each site via clinician-based assessment [32].

### Train-test split procedure

For the test and train split procedure, the total number of subjects (9,658) was split into two groups: (a) subjects from samples including GAD cases and controls, and (b) subjects from samples composed of only controls. The latter was used as the training sample, which consisted of 3,511 individuals (Table 1). Ten percent of the training sample was used to track model performance while training (referred in the results as *validation sample*). The test sample (n=6,147; Table 2) consisted of data from studies that contained subjects with GAD and controls.

### Brain age model

The present analysis used the PyTorch library for the creation of the CNN model [51]. The input to the network was a 5D array consisting of batch size (the number of subjects given to the model at a time for training) by channels by X image dimension by Y image dimension by Z image dimension. The four channels were the gray and white matter segmentations, as well as the T1-weighted image and Jacobian images, all in MNI152 space.

**Figure 1:**
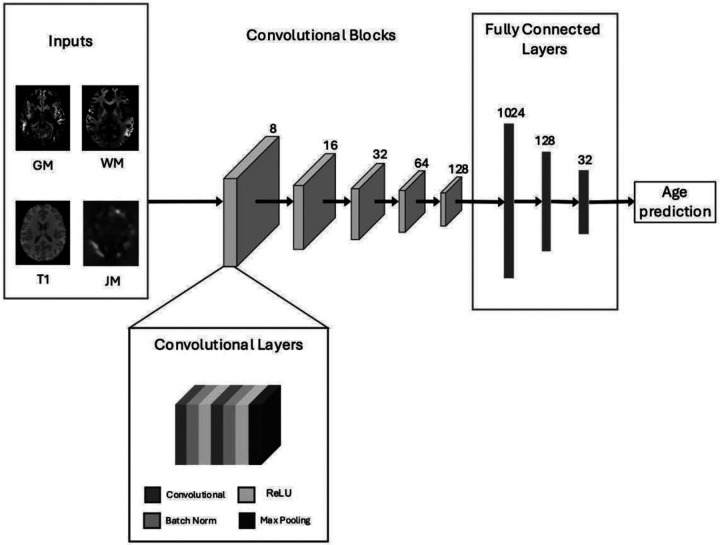
Overview of the CNN Model Architecture. *Note*. The CNN architecture was designed with four input channels for gray and white matter segmentations, as well as the T1-weighted and Jacobian images. The model consists of five repeated convolutional blocks, each containing 3D convolutional layers, batch normalization, ReLU activations and max pooling. The number of filters used in each block are denoted above. After the last convolutional block, the model includes a flattened layer.

The network architecture includes five repeated convolutional blocks, each containing 3D convolutional layers, batch normalization, ReLU activations and max pooling (Figure 1). This block was repeated five times, at each step doubling the number of convolutional filters but halving the size of the image via the max pooling layer. Thus, the number of filters was 8 for the first layer, and the final number of filters was 128. We used a 3×3×3 kernel with padding of size 1. Max pooling layers had a stride distance of 2 to downsize each dimension image by half. Layers following batch normalization utilized a ReLU activation function. After the last block, the image was flattened to a 1,024 size fully connected dense layer and then connected to two more fully connected layers of size 128 and 32 before connecting to the output node where age predictions were made with a ReLU function. Between each fully connected layer, a dropout layer was added with a rate of 50 percent, as is recommended for CNNs [52]. The total number of model parameters was 1,020,721.

The MAE was measured across each batch to track model performance and as the model loss function. The mean squared error (MSE) was also tracked but not used to optimize model performance. The Adaptive Moment Estimation (Adam) optimizer was used to update the model weights [53]. Gradient norm clipping was used to prevent exploding gradients [54], which occurs when the gradients of the model parameters become excessively large during training, leading to instability. The maximum gradient value was set to 1 and learning rate was set to 0.0001.

A homogeneous ensemble strategy was used to combine the predictions of multiple models [55]. Other machine learning prediction work has used ensemble strategies with deep learning models [55, 56], including brain age prediction [57], which has been found to generate better predictive results. Since neural networks may converge to several possible local minima, it is ideal to use strategies that provide stable results. Instability in results would complicate downstream analysis and interpretation of results generated by a CNN. As is recommended when using homogeneous ensembles, it is best practice to introduce randomness into the constituent models, usually in the form of a diversified training set [55]. An ensemble of eight model instantiations was created for the present analysis. Each instantiation was initialized with random weights, but the architecture for each was identical. Ten percent of the training data was randomly selected to serve as the validation data for each model, such that each model in the ensemble had a different set of 90% of the full training sample. After the training process, the eight models’ age predictions for each subject in the test set were then averaged together. These averaged predictions were treated as the overall predicted ages to be used in subsequent analyses and subtracted from the subject’s true chronological age to generate the overall PAD for all participants within the entire test set.

### Statistical Analysis

All statistical tests were conducted in PALM - Permutation Analysis of Linear Models [58]. The PAD measure was entered as the dependent variable for all models. Three general linear models (GLMs) were tested (Supplementary Table 3): one was a simple model with only the diagnosis as the variable of interest, the second was a model with diagnosis and covariates (age, sex, scanner, comorbidities, and medication), and a third model with the same covariates, but with the interaction between diagnosis and age as the variable of interest. All models included scanner as a covariate and allowed each site to have their own parameter estimates (slopes and intercepts), thus accommodating between-site differences, further dispensing with the need for explicit harmonization, and avoiding that larger sites would dominate the results. Variance groups [58, 59] were used to allow different variances between GAD and controls. The number of permutations (sign-flippings) was set at 10,000. All results were corrected for multiple testing using the familywise error rate (FWER).

Levene’s test for equal variance was used to investigate if participants with GAD have a higher variance in the PAD than HCs. Levene’s test was also used to examine the difference across diagnosis and in adults compared to children and adolescents (as preregistered with a threshold of 25 years. This was chosen a priori as an appropriate age for the transition from adolescence to young adulthood). Of note, we do not conduct the exploratory analysis on symptoms scale as described in the preregistration, as we unfortunately did not have enough symptoms scale data at the time of this analysis.

## Results

**Figure 3:**
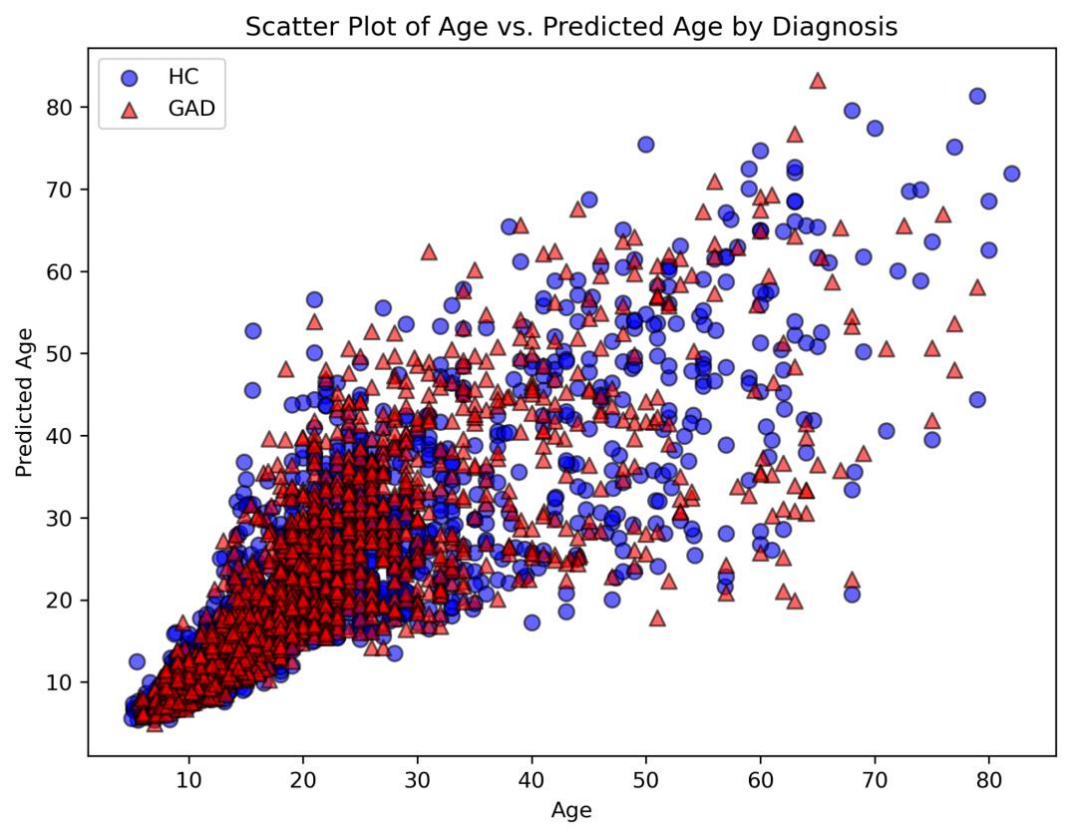
Scatter Plot of True Age vs. Predicted Age Sorted By Diagnostic Status

The MAE for the training set was 2.95 years. The MAE for the validation set was 2.30 years. Figure 3 displays a scatterplot of the predicted ages with respect to the true ages in the testing set. Averaged across models in the ensemble, the testing set had an overall MAE of 3.80 years, 2.94 years for control participants, and 6.27 years for subjects with a GAD diagnosis (Supplemental Table 2). A key consideration was to establish the extent to which each model generated consistent predictions and evaluation metrics. Testing results across models revealed stable performance. This was determined by using intraclass correlation (ICC) of the form [2,K] to measure the reliability of the age predictions [60]. To test the reliability of each model’s generated age prediction, ICC was calculated across these predictions. The ICC for the predicted age was 0.99, indicating excellent reliability across models. Table 3 displays the results of the MAE and PAD across samples. The MSE for the overall sample was 47.77, whereas the MSE for the control group was 31.72 and for the GAD group 93.57.

Once the average brain age predictions were subtracted from chronological age, the overall PAD was 0.49 years, with a standard deviation of 6.10. For controls, the predicted age difference was 0.36 years (SD = 4.97). For GAD subjects, the PAD was .87 years (SD = 8.53). None of the three GLM models established a statistically significant relationship between PAD and diagnosis (all *p* > .05). This suggests that none of the independent variables (age, sex, scanner, comorbidities, and medication) had any meaningful relationship with the PAD across any of the sites. True age and predicted age significantly correlated (*r*=.86, p<.001), as did true age and PAD (*r*=−.26, p<.001). The greater MAE of subjects with GAD relative to controls was noteworthy.

**Figure 4:**
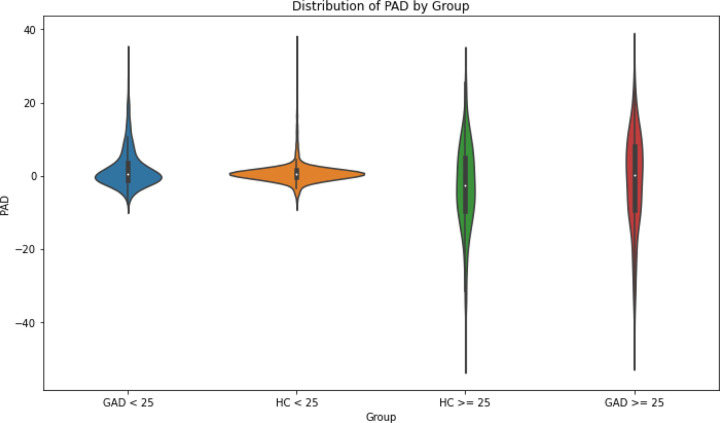
Violin Plot of Variance in PAD across Age and Diagnostic Groups

Levene’s test of equality of variances suggests a statistically significant difference in the variance of PAD between controls and subjects with GAD (*W* = 442.98, *p* < .001), with GAD subjects presenting more variance compared to healthy controls (GAD SD: 8.53, controls SD: 4.97). To examine if age plays a role in the degree of variance between those with and without GAD, the test was implemented after partitioning the data into four groups: healthy volunteers under age 25, individuals with GAD under 25, healthy volunteers over age 25, and individuals with GAD over 25. Subjects with GAD had greater variance, with those over 25 having the greatest variance for all groups. Full results for these tests are shown in Table 4 and 5. A violin plot illustrating the degree of variance in each group is displayed in Figure 4. Mean, median, and standard deviation of the different age and diagnostic groups are shown in Supplemental Table 4.

T tests between the mean ages of individuals with and without GAD were conducted between all sites to examine any significant differences. Several sites were found to have statistically significant differences in age (listed in Table 2). This difference was also significant in the overall sample. Due to these results, we conducted several supplementary analyses, with only the ENIGMA-ANX sites (excluding ABCD, CMI and BHRC) in an attempt to age match the subjects across diagnostic groups. A T test demonstrated that once these sites were removed, both subjects with and without GAD were age matched. With these sites removed, we reanalyzed the same three GLM models with these new samples. As before, none of these models established a statistically significant relationship between PAD and diagnosis (all *p* > .05). Additionally, we also re-examined the variance in PAD across age and diagnostic groups. The effect of greater variance in subjects with GAD, and greater variance in GAD subjects above 25 was replicated (See supplemental tables 4 and 5).

## Discussion

The current study used a CNN to estimate brain age and compute the PAD across individuals with GAD and controls. We used data from 3,500+ healthy individuals across the lifespan to train our model and then tested the model on 6,000+ independent individuals with and without GAD. The difference in PAD between individuals with GAD and controls was then investigated. With respect to our first hypothesis, we did not find a significant difference in PAD between individuals with GAD and controls. However, consistent with our second hypothesis, we found that individuals with GAD have greater PAD variability, particularly in those older than 25 years. After the PAD for each subject was generated, we conducted secondary analyses to examine (1) the relation of diagnostic status across sites, (2) how medication and comorbid disorders relate to PAD, and (3) interaction of diagnosis by age. All three secondary analyses showed no significant relation between the variables of interest and PAD, even after age matching subjects.

Our findings are consistent with prior work showing no significant differences in PAD between GAD and controls and with dimensional measures of anxiety in youth [22]. However, these findings contrast with work within anxiety disorders broadly that found a positive PAD in a substantially smaller sample consisting of participants with GAD, PD and SAD diagnoses, when accounting for medication effect [18], and other work finding a greater PAD in adolescents with GAD [22]. The heterogeneity of GAD in its clinical presentation, may explain portions of these inconsistent findings.

Although the group difference findings were non-significant, the significantly higher variance in the GAD group is noteworthy, as heterogeneity within psychopathology is a common finding [61, 62]. Other brain age analyses of psychiatric disorders have also found greater variability in the clinical population compared to healthy controls. Han et al. [17] found higher variability among individuals with depression than in healthy individuals. For healthy controls, the MAE was 6.50 and 6.84 years for males and females, respectively. Similarly, within the MDD group, the MAE was 6.72 for males and 7.18 for females. In a replication study, the same authors found that males in the control group had an MAE of 6.85 and females had 7.84. While in the depression group, males showed a larger MAE of 7.57 and females had 9.24, again suggesting greater variability within the clinical population. The same trend appeared in another study examining depression and anxiety disorders [18], where healthy controls manifested an MAE of 5.97, but those in the disorder group had an MAE of 6.73. Finally, Tønnesen et al. [29] found that healthy controls had a MAE of 6.92, patients with bipolar disorder had an MAE = 6.85, and patients with schizophrenia had an MAE = 7.11. This could suggest potentially greater variability in subjects with schizophrenia, but similar variability in bipolar disorder to healthy controls. While notable, these differences in past research were not reported as main findings, nor statistically tested. However, the present study used a statistical procedure to establish a statistically significant difference in variance across those with GAD and controls; this suggests a further avenue for research in brain age prediction for psychiatric disorders. The higher variance in the GAD group indicated that the model performs more poorly in this population. Further investigating the sources of increased variance in the patient population might help understand new mechanisms for the development of psychiatric disorders.

Given that there was a statistically significant age difference in the subjects with GAD, we conducted further supplemental analysis with the non-ENIGMA samples removed to ensure subjects were age matched. This new GLM did not suggest an effect of advanced aging or any other variables having a relationship to the PAD. However, the effect of larger variance in the PAD for individuals with GAD replicated in the age matched sample. This means that the inclusion of sites such as ABCD, where large number of subjects with narrowly distributed ages did not drive the differences in variances between these groups.

The observation of greater PAD variability in individuals with GAD, despite no overall shift in mean brain age, has meaningful implications. Clinically, it underscores the heterogeneity of GAD and raises the possibility that subgroups of patients may experience neurostructural changes that are obscured when averaging across the diagnostic group (e.g., endophenotypes). This possibility, if true, would partially explain the mixed findings in the literature and raises the possibility that PAD variance could represent a transdiagnostic marker of risk or resilience. Moreover, the finding of greater PAD variability in individuals over 25 suggests that brain aging trajectories in GAD may become more divergent in adulthood, highlighting the need for longitudinal research to map the neurostructural progression of the disorder over time. Future studies should explore whether increased PAD variability is associated with symptom chronicity, cognitive decline, treatment response, or comorbidities.

The results of this study should be viewed in the context of several limitations. First, although this study has a large sample size, there was an uneven age distribution across the samples, with a predominance of younger participants largely due to the inclusion of the ABCD data. Additionally, PAD was related to true age, which is a common issue in brain age studies [63, 64]; to address this we included age as a covariate, with no significant results. A second limitation is that, while we constructed our model based on the best existing literature and practices on CNN, this area of research continues to develop quickly. The proposed model does not exhaust possible architectures for brain age prediction, or even all possibilities among CNN models. It should also be noted that the use of a whole-brain VBM approach in combination with the CNN architecture does not allow for group comparisons of structural changes in specific regions of interest (ROI) previously associated with GAD, as is common practice in the neuroimaging literature. However, the CNN approach demonstrates more accurate overall brain age estimations compared to linear ROI approaches in healthy subjects [27]. Thus, the CNN architecture is more sensitive to subject level spatial information making it a better model choice for the proposed analyses with the downside of lacking the granularity of an ROI-based linear approach.

In summary, we found no significant difference in PAD between individuals with GAD compared to healthy controls. This is consistent with prior work in adolescents with anxiety symptoms that found no association between PAD and anxiety. We found PAD was not associated with medication use or comorbid disorders related to PAD, and found no interaction between age and diagnostic status with PAD. In other words, the results in this analysis do not support the hypothesis that GAD is associated with accelerated brain aging. Of note, we did find significantly larger variability in PAD for GAD compared to the healthy controls. This difference in variability between a psychiatric group and controls is an area that should be more robustly examined and reported in future studies of PAD. Lastly, the training and testing samples assembled for this study are substantially larger than previous work within anxiety domains and brain PAD. Reporting these null results from a large mega-analysis is important as it may illustrate that a more detailed approach is necessary to fully capture the heterogeneity underlying GAD. It might be that specific subpopulations, treatment exposures, or specific symptoms are associated with differences in brain age; however, it is difficult to disentangle these without harmonized data collection.

## Supplementary Material

1

## Figures and Tables

**Figure 2:**
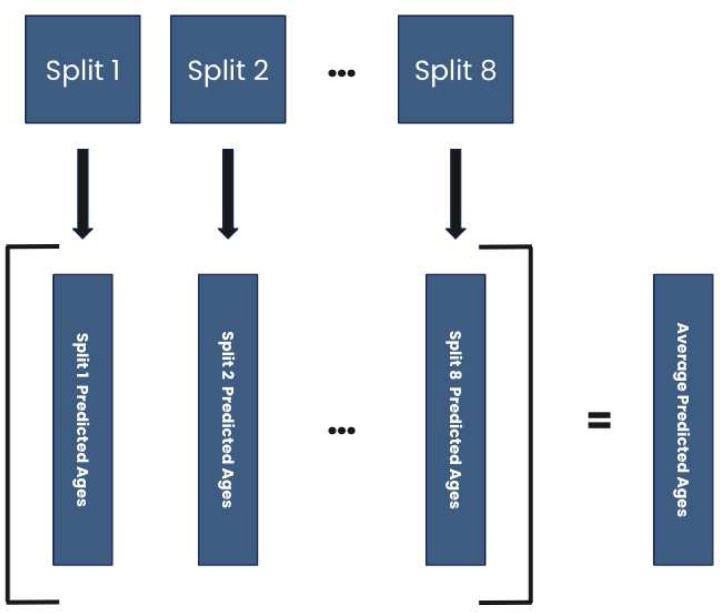
Model Ensemble Procedure *Note*. Procedure for generating average predictions for the model ensemble. Eight splits of the training data were created, which used a different 90/10% split of training to validation data. Each of these splits was given to a unique instantiation of the model with identical architecture but instantiated with random weights.

**Table 1:** Number of Subjects and Descriptive Statistics - Training Sample

Site	Total n	Average Age	Standard Deviation	Age Min	Age Max
HCP	412	28.71	3.67	22	37
HCP Development	612	14.52	4.04	5.58	21.92
OASIS	334	68.93	9.29	43	93
PING	588	12.42	5.12	3.08	21.67
PNC	1,565	15.02	3.66	8.08	23.08
Total/Average	3,511	21.23	16.85	16.35	39.33

HCP: Human Connectome Project, HCP Development Human Connectome Project-Development, OASIS: Open Access Series of Imaging Studies, PING: Pediatric Imaging, Neurocognition, and Genetics study. PNC: Philadelphia Neurodevelopmental Cohort

**Table 2:** Number of Participants and Descriptive Statistics - Testing Sample

Site	Total n	Age Min	Age Max	HC Sample	GAD Sample	GAD Average Age (SD)	HC Average Age (SD)	*p*
ABCD	2,857	8.91	11	2,778	79	10.05 (.62)	9.92 (.62)	.07
Barcelona	69	18	40	46	23	23.61 (4.3)	21.71 (4.48)	.10
Baylor	226	12	79	128	98	28.84 (9.18)	24.39 (12.84)	**<.05**
BHRC	767	7.02	21.37	458	309	13.38 (3.15)	11.70 (2.64)	**<.05**
Boystown	95	13	18	45	50	15.82 (1.35)	15.44 (1.62)	.23
Chicago-Milad	43	18	59	16	27	30.25 (9.71)	32.37 (12.96)	.58
Chicago-Phan	141	18	60	41	100	26.36 (8.51)	25.29 (9.75)	.54
Cincinnati	20	12	18	11	9	13.66 (2.05)	14.18 (1.89)	.59
CMI-HBN	185	5.01	21.36	97	88	12.24 (3.53)	9.57 (3.21)	**<.05**
Dresden	93	18	51	46	47	30.23 (9.94)	28.47 (7.90)	.35
Duke	40	6	10	19	21	7 (1.06)	7.47 (.99)	.16
SHIP	32	41	70	22	10	55.9 (7.95)	57.72 (8.01)	.57
Harvard	237	18	40	51	186	24.64 (4.31)	24.68 (3.77)	.95
Houston	263	8	68.23	251	12	16.69 (9.02)	29.21 (14.39)	**<.05**
Milan	91	20.89	72.57	61	30	44.31 (14.91)	34.11 (12.42)	**<.05**
Muenster	53	19	56	29	24	29.45 (10.01)	27.82 (9.04)	.54
Pittsburgh-Andreescu	64	19	82	26	38	57.78 (16.51)	56.84 (20.22)	.84
Pittsburgh-Price	69	18	54	0	69	N/A	N/A	N/A
PROTAIA	43	13	22	25	18	16.67 (2.26)	17.80 (2.54)	.14
Rome	38	18	55	20	18	29.55 (6.92)	29.15 (9.18)	.88
San Raffaele	89	23	63	69	20	44.65 (10.61)	45.26 (10.97)	.83
SDAN	295	8.1	51.14	156	139	12.77 (2.78)	13.70 (4.37)	**<.05**
SNFA	60	19	50	38	22	31.27 (9.97)	29 (7.17)	.36
Stony Brook	60	18	49	19	41	22.87 (5.91)	21.57 (5.74)	.43
UCSD	91	17	53	47	44	28.36 (10.75)	23.40 (8.27)	**<.05**
Wash U	63	8	12	32	31	9.96 (1.44)	9.90 (1.15)	.85
IOL	63	19	71	21	42	39.92 (13.38)	41.19 (16.17)	.76
Total/Average	6,147	15.66	46.58	4,552	1,595	22.16 (13)	14.53 (10.59)	**<.05**

GAD: generalized anxiety disorder, HC: healthy controls, p: p value, ABCD: Adolescent Brain Cognitive Development Study, BHRCS: Brazilian High Risk Cohort Study, CMI-HBN: Child Mind Institute Healthy Brain Network, IOL: Institute of Living, HCP: Human Connectome Project, HCPdev: Human Connectome Project Development, PING: Pediatric Imaging, Neurocognition, and Genetics Study, OASIS: Open Access Series of Imaging Studies, PNC: Philadelphia Neurodevelopmental Cohort, PROTAIA: Anxiety Disorders Program for Child and Adolescent Psychiatry, SDAN: Section on Development and Affective Neuroscience, SNFA: Section on Neurobiology of Fear and Anxiety, UCSD: University of California – San Diego, WashU: Washington University. SHIP: Study of Health in Pomerania.

**Table 3:** Testing Set Age, Predicted Age, and PAD Organized by Sample

	GAD n	GAD PAD Mean	GAD PAD SD	HC n	HC PAD Mean	HC PAD SD
ABCD	79	0.179	1.18	2,778	0.58	1.19
Barcelona	23	−1.45	6.03	46	−1.83	4.79
Baylor	98	5.36	7.71	128	1.06	5.40
BHRC	309	1.60	4.92	458	0.85	4.53
Boystown	50	−2.21	2.18	45	−2.92	1.72
Chicago-Milad	27	4.31	8.52	16	4.89	8.97
Chicago-Phan	100	.94	5.95	41	1.11	7.38
Cincinnati	9	−1.14	0.96	11	−0.88	1.29
CMI	88	2.22	4.03	97	1.20	2.32
Dresden	47	9.23	7.01	46	10.92	7.41
Duke	21	0.23	1.33	19	−0.48	0.91
SHIP	10	6.25	6.03	22	1.69	7.17
Harvard	186	4.76	6.09	51	4.43	5.36
Houston	12	−.2.67	5.44	251	−4.61	8.16
Milan	30	−7.04	9.98	61	−8.46	7.63
Muenster	24	12.1	8.72	29	9.04	8.96
Pittsburgh-Andreescu	38	−23.27	11.46	26	−19.83	12.25
Pittsburgh-Price	69	−8.51	5.82	N/A	N/A	N/A
PROTAIA	18	6.51	5.90	25	8.72	8.64
Rome	18	5.24	7.49	20	3.72	7.63
San Raffaele	20	11.65	4.99	69	5.41	8.72
SDAN	139	−0.19	1.42	156	0.14	2.41
SNFA	22	1.58	8.56	38	−0.81	9.93
Stony Brook	41	9.26	7.46	19	12.76	6.74
UCSD	44	−3.09	9.28	47	−0.99	6.33
Wash U	31	2.42	1.13	32	2.47	1.21
IOL	42	−14.80	8.64	21	−13.17	9.31

PAD predicted age difference, SD standard deviation, GAD generalized anxiety disorder, HC healthy controls, ABCD: Adolescent Brain Cognitive Development Study, BHRCS: Brazilian High Risk Cohort Study, CMI-HBN: Child Mind Institute Healthy Brain Network, IOL: Institute of Living, HCP: Human Connectome Project, HCPdev: Human Connectome Project Development, PING: Pediatric Imaging, Neurocognition, and Genetics Study, OASIS: Open Access Series of Imaging Studies, PNC: Philadelphia Neurodevelopmental Cohort, PROTAIA: Anxiety Disorders Program for Child and Adolescent Psychiatry, SDAN: Section on Development and Affective Neuroscience, SNFA: Section on Neurobiology of Fear and Anxiety, UCSD: University of California – San Diego, WashU: Washington University. SHIP: Study of Health in Pomerania

**Table 4:** Characteristics of PAD by Diagnosis and Age

Group	Mean PAD	Median PAD	Standard Deviation	Variance
GAD < 25	1.86	0.42	5.25	27.64
HC < 25	0.77	0.51	3.01	9.06
GAD > 25	−1.42	0.10	13.08	171.136
HC > 25	−2.86	−2.70	11.57	134.04

**Table 5:** Levene’s Tests Comparing Variances in PAD across Diagnostic and Age Groups

Group 1	Group 2	W	P
GAD < 25	HC < 25	348.52	<.001
GAD < 25	GAD > 25	489.6	<.001
GAD < 25	HC > 25	394.01	<.001
HC < 25	GAD > 25	2541.49	<.001
HC < 25	HC > 25	2221.09	<.001
GAD > 25	HC > 25	6.74	<.001

## Data Availability

The data analyzed in this study is a combination of open datasets and the collaborative work of ENIGMA-Anxiety. The ENIGMA-Anxiety Working Group accepts research proposals from *bona fide* researchers. The data are shared by each participating PI after approval of the project. For more information please see: https://enigma.ini.usc.edu/ongoing/enigma-anxiety/. The code used in the analysis described here will be available at https://github.com/coreyjr2/ENIGMA_BrainAge_GAD_structural.
