# Supplementary material for: Brain Age Prediction in Generalized Anxiety Disorder using a Convolutional Neural Network
Source: Res Sq. 2025 Jul 1:rs.3.rs-6866544. Preprint. [Version 1] doi: 10.21203/rs.3.rs-6866544/v1 (PMC12236912; doi:10.21203/rs.3.rs-6866544/v1)
Supplement: 1 [file NIHPPRS6866544V1-supplement-1.pdf]

Supplementary Material

Supplemental Table 1

Sample Selection Procedure

Site	Subjects with VBM	After data entry exclusions	After QC (Final Sample)
ABCD	2,994	2,994	2,857
Barcelona	91	91	69
Baylor	228	228	226
BHRC	799	797	767
Boystown	95	95	95
Chicago-Milad	43	43	43
Chicago-Phan	147	147	141
Cincinnati	20	20	20
CMI	198	198	185
Dresden	94	94	93
Duke	40	40	40
SHIP	36	36	32
Harvard	260	260	237
HCP	414	414	412
HCPdev	652	652	612

Houston	273	273	263
Milan	97	97	91
Muenster	54	54	53
OASIS	480	480	334
PING	741	729	588
Pittsburgh-Andreescu	79	79	64
Pittsburgh-Price	69	69	69
PNC	1,597	1,597	1,565
PROTAIA	44	44	43
Rome	40	40	38
San Raffaele	92	92	89
SDAN	295	295	295
SNFA	63	63	60
Stony Brook	61	61	60
UCSD	96	96	91
Wash U	63	63	63
IOL	64	64	63
Total	10,319	10,305	9,658

VBM: Voxel Based Morphometry, QC: quality control, GAD: generalized anxiety disorder, HC: healthy controls, ABCD: Adolescent Brain Cognitive Development Study, BHRCS: Brazilian High Risk

Cohort Study, CMI-HBN: Child Mind Institute Healthy Brain Network, IOL: Institute of Living, HCP: Human Connectome Project, HCPdev: Human Connectome Project Development, PING: Pediatric Imaging, Neurocognition, and Genetics Study, OASIS: Open Access Series of Imaging Studies, PNC: Philadelphia Neurodevelopmental Cohort, PROTAIA: Anxiety Disorders Program for Child and Adolescent Psychiatry, SDAN: Section on Development and Affective Neuroscience, SNFA: Section on Neurobiology of Fear and Anxiety, UCSD: University of California – San Diego, WashU: Washington University. SHIP: Study of Health in Pomerania

Supplemental Table 2

Model Performance by Each Model in the Ensemble

	Total Test MAE	HC Test MAE	GAD Test MAE
Model 1	3.70	2.85	5.97
Model 2	3.57	2.80	6.46
Model 3	4.25	3.22	5.61
Model 4	3.33	2.66	5.66
Model 5	3.28	3.29	6.63
Model 6	4.31	3.29	6.45
Model 7	4.14	3.06	5.94
Model 8	3.82	2.91	5.87
Overall	3.80	2.94	6.27

MAE: mean absolute error. HC: healthy controls, GAD: generalized anxiety disorder.

Supplemental Table 3

Overview of variables and contrasts of interest in the secondary analysis.

Dependent Variable	Independent Variables			
		**Model 1 (group comparison)**	**Model 2 (Covariates)**	**Model 3 (Moderation)**
Predicted Age Difference (PAD)	Variables of interest	Diagnosis	Diagnosis	Diagnosis
	Nuisance Variables		Age<br>Age^2<br>Scanner<br>Medication<br>Comorbid SAD, PD, AG, SPH, other anxiety disorders, MDD, OCD, PTSD, SUD, other disorders	Age<br>Age^2<br>Scanner<br>Medication<br>Comorbid SAD, PD, AG, SPH, other anxiety disorders, MDD, OCD, PTSD, SUD, other disorders<br>Age * Diagnosis

PAD: predicted age difference, SAD: separation anxiety disorder, PD: panic disorder, AG: agoraphobia, SPH: specific phobia, MDD: major depressive disorder, OCD: obsessive compulsive disorder, PTSD: post-traumatic stress disorder, SUD: substance use disorder

Supplemental Table 4

Descriptive statistics of predicted age difference by age and anxiety disorder status with ABCD, CMI, and BHRC removed.

Group	Mean	Median	Standard Deviation	Variance
GAD < 25	2.14	0.61	5.80	33.67
HC < 25	1.45	0.13	5.64	31.85
GAD > 25	-1.42	-2.70	11.57	171.13
HC > 25	-2.86	-2.70	11.57	134.04

Supplemental Table 5

Levene's Tests Comparing Variances in PAD across Diagnostic and Age Groups with ABCD, CMI, and BHRC removed

Group 1	Group 2	W	P
GAD < 25	HC < 25	10.71	<.001
GAD < 25	GAD > 25	436.01	<.001
GAD < 25	HC > 25	346.86	<.001
HC < 25	GAD > 25	586.32	<.001
HC < 25	HC > 25	481.04	<.001
GAD > 25	HC > 25	6.74	<.001
